# Structural Basis for Binding of Allosteric Drug Leads in the Adenosine A_1_ Receptor

**DOI:** 10.1038/s41598-018-35266-x

**Published:** 2018-11-15

**Authors:** Yinglong Miao, Apurba Bhattarai, Anh T. N. Nguyen, Arthur Christopoulos, Lauren T. May

**Affiliations:** 1 Center for Computational Biology and Department of Molecular Biosciences, University of Kansas, Lawrence, KS 66047 USA; 2 Drug Discovery Biology, Monash Institute of Pharmaceutical Sciences and Department of Pharmacology, Monash University, Parkville, VIC 3052 Australia

## Abstract

Despite intense interest in designing positive allosteric modulators (PAMs) as selective drugs of the adenosine A_1_ receptor (A_1_AR), structural binding modes of the receptor PAMs remain unknown. Using the first X-ray structure of the A_1_AR, we have performed all-atom simulations using a robust Gaussian accelerated molecular dynamics (GaMD) technique to determine binding modes of the A_1_AR allosteric drug leads. Two prototypical PAMs, PD81723 and VCP171, were selected. Each PAM was initially placed at least 20 Å away from the receptor. Extensive GaMD simulations using the AMBER and NAMD simulation packages at different acceleration levels captured spontaneous binding of PAMs to the A_1_AR. The simulations allowed us to identify low-energy binding modes of the PAMs at an allosteric site formed by the receptor extracellular loop 2 (ECL2), which are highly consistent with mutagenesis experimental data. Furthermore, the PAMs stabilized agonist binding in the receptor. In the absence of PAMs at the ECL2 allosteric site, the agonist sampled a significantly larger conformational space and even dissociated from the A_1_AR alone. In summary, the GaMD simulations elucidated structural binding modes of the PAMs and provided important insights into allostery in the A_1_AR, which will greatly facilitate the receptor structure-based drug design.

## Introduction

G-protein-coupled receptors (GPCRs) are key cellular signaling proteins and represent primary targets of ~1/3 of currently marketed drugs^1^. Four GPCR subtypes, the A_1_, A_2A_, A_2B_ and A_3_ receptors, mediate the effects of adenosine, an endogenous nucleoside modulator that plays a critical role in cytoprotective function^2^. In particular, preclinical studies suggest the adenosine A_1_ receptor (A_1_AR) is an important novel drug target for treating human diseases such as ischemia-reperfusion injury and neuropathic pain^3^. However, the high conservation of the endogenous agonist binding (“orthosteric”) site across the different adenosine receptor subtypes, has hindered the therapeutic development of A_1_AR agonists due to off-target side effects^4^. Furthermore, since the A_1_AR is expressed in different human tissues, including the heart and brain, traditional agonists can also cause on-target side effects. An alternative strategy involves the development of positive allosteric modulators (PAMs), which through binding to a topographically distinct (“allosteric”) site on the receptor, can increase the responsiveness of A_1_AR to endogenous adenosine within the local regions of its elevated production. PAMs have the potential to overcome the current limitations associated with orthosteric agonists and thus are a promising approach for the development of subtype selective A_1_AR therapeutics that are not associated with unwanted effects^5–10^.

The first A_1_AR PAM, PD81723, was identified by Bruns and coworkers in 1990^11,12^. Since then, a number of research groups have performed extensive structure-activity relationship (SAR) studies with the aim to improve the compound pharmacology and chemical properties^11,13–21^. Generally, the success of SAR studies has been limited due to a lack of structural basis for chemical modifications of the reference compound PD81723. For instance, heterocycles were designed to replace the phenyl group at the 3-position of the thiophene ring in PD81723 to increase solubility and improve binding affinity through a predicted hydrogen bond with the A_1_AR allosteric site, unfortunately the synthesized derivatives were found to be less potent than PD81723^22^. However, despite the limitations, SAR programs have yielded PD81723 derivatives with improved pharmacology including the T62^23,24^ and VCP171^25^. Notably, T62, evaluated by King Pharmaceuticals progressed to Phase IIB clinical trial (for neuropathic pain), but failed due to lack of efficacy^23,24^. Overall, these compounds still suffer from major limitations for pharmaceutical use, such as low solubility, affinity and cooperativity.

To date, structural information regarding PAM interactions with the A_1_AR has been largely unavailable to guide previous drug design efforts aimed towards the development of therapeutically effective A_1_AR PAMs^5^. Mutagenesis and molecular modeling studies have suggested that the A_1_AR allosteric site may reside within the second extracellular loop (ECL2)^26,27^, however the precise location of the allosteric site and the molecular mechanisms underlying the allosteric modulation of PD81723 and other PAMs remain unclear. Recently, the first X-ray crystal structure of the A_1_AR (PDB: 5UEN) was determined by Christopoulos and coworkers^28^. In the structure, the A_1_AR was bound to an irreversible antagonist DU172, which forms a covalent bond with Tyr271^7.36^ in the transmembrane (TM) helix 7; superscript denoting Ballesteros-Weinstein residue numbering^29^. Compared with previous X-ray structures of the A_2A_AR^30,31^, the A_1_AR exhibits a significantly wider extracellular cavity with a distinct conformation of the ECL2. Another similar X-ray structure was determined for the A_1_AR bound by the antagonist in the inactive state^32^. The X-ray structures serve as an excellent starting point for computational modeling and structure-based drug discovery of the A_1_AR.

Molecular dynamics (MD) is a powerful computational technique for simulating biomolecular dynamics on an atomistic level^33^. For GPCRs, MD has been applied to simulate binding of both orthosteric and allosteric ligands^28,34,35^. Using the specialized supercomputer Anton, Dror *et al*. performed microsecond-timescale MD simulations on the β_1_ and β_2_ adrenergic receptors (β_1_AR and β_2_AR)^34^. These simulations showed that antagonist and agonist ligands entered the receptor orthosteric site through an opening between ECL2-ECL3, which was suggested to be a dominant binding pathway of GPCR drugs. Subsequent Anton MD simulations captured the same orthosteric ligand binding pathway for the M_2_ and M_3_ muscarinic acetylcholine GPCRs (mAChRs)^28^. Binding of several known negative allosteric modulators (NAMs) to the extracellular vestibule of the M_2_ receptor was observed in further Anton MD simulations^35^. The modulators formed cation-π interactions with aromatic residues in the receptor extracellular vestibule. The extracellular allosteric binding mode was confirmed by mutation experiments and later by the X-ray structure of the active M_2_ receptor that is recognized by a PAM^36^. Despite these successes, direct MD simulations are computationally expensive for studying protein-ligand binding. They often suffer from insufficient sampling of slow ligand binders^28^ and cannot capture ligand dissociation due to limited simulation timescales.

During the last several decades, many enhanced sampling methods have been developed to improve MD simulations^37–43^. Among these methods, metadynamics^44,45^, random acceleration MD^46,47^, steered MD^48^, temperature accelerated MD (TAMD)^28,49^, accelerated MD (aMD)^50,51^ and Gaussian aMD (GaMD)^52–54^ have been applied to simulate ligand binding to GPCRs^55^. Metadynamics was applied to simulate binding of a PAM to the δ-opioid receptor in the presence of an agonist^56^ and calculate ligand binding free energies in the β_2_AR^57^. We performed aMD simulations on binding of the tiotropium antagonist, acetylcholine agonist and arecoline partial agonist to the M_3_ muscarinic receptor^58^. In comparison with the previous Anton MD simulations^28^, aMD captured a similar ligand binding pathway, but with significant speedup (~80 times faster for agonist binding to the receptor orthosteric ligand-binding site). Using GaMD that provides unconstrained enhanced sampling and improved free energy calculations^52–54^, we also captured spontaneous binding of the agonist acetylcholine and identified its low-energy binding sites in the M_3_ receptor^53^. The energetically preferred pathway of agonist binding identified from the GaMD simulation was similar to that found in previous long-timescale cMD^34^ and aMD^58^ simulations. Furthermore, we successfully applied GaMD to capture both dissociation and binding of the arecoline partial agonist in the M_2_ receptor^59^. Therefore, GaMD is well suited for investigating ligand binding of large biomolecules such as GPCRs.

In this study, we have applied GaMD to simulate binding of allosteric drug leads to the A_1_AR. Extensive GaMD simulations using the AMBER and NAMD simulation packages at different acceleration levels captured spontaneous binding of two prototypical PAMs to the A_1_AR. The GaMD simulations also allowed free energy calculations to identify low-energy binding modes of the PAMs at the putative allosteric site formed by ECL2 of the receptor, which is highly consistent with the mutation experimental data. Furthermore, PAM binding was found to stabilize agonist binding at the receptor orthosteric site. Therefore, GaMD simulations have provided a greater understanding of the structural binding modes and allosteric effects of PAMs at the A_1_AR.

## Results

### GaMD simulations captured spontaneous binding of PAMs

**Figure 1:**
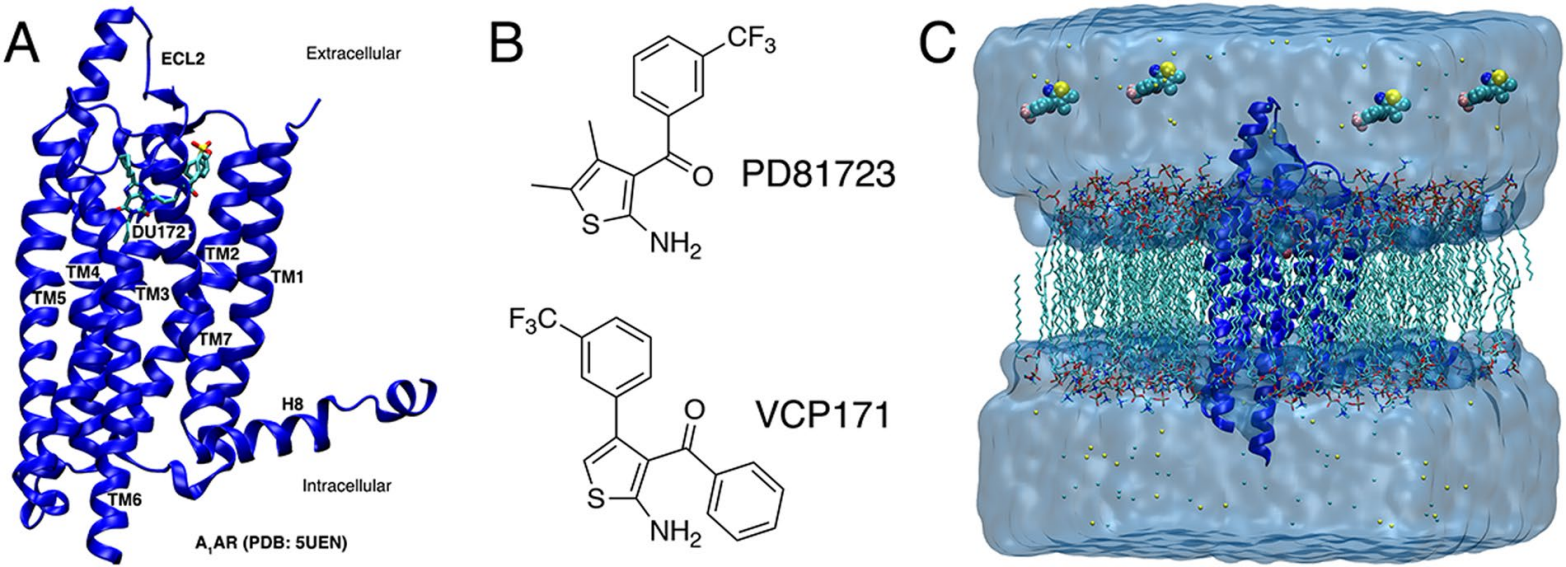
(**A**) X-ray structure of the DU172 antagonist-bound adenosine A_1_ receptor (A_1_AR) (PDB: 5UEN), (**B**) the structure of the two prototypical A_1_AR positive allosteric modulators (PAMs), PD81723 and VCP171, used in this study and (**C**) computational model used for the simulations. The receptor was inserted into a POPC lipid bilayer and solvated in an aqueous medium of 0.15 M NaCl. After removal of antagonist from the A_1_AR X-ray structure, NECA was placed in the orthosteric pocket with atomic coordinates copied from the A_2A_AR X-ray structure (PDB: 2YDV) after aligning the two receptor transmembrane domains. Four molecules of each PAM were placed >20 Å away from the receptor.

Using the first X-ray crystal structure of the A_1_AR (PDB: 5UEN, Fig. 1A)^60^, we have performed all-atom GaMD simulations to investigate binding of two prototypical PAMs, PD81723^11,12^ and VCP171^25^ (Fig. 1B). The antagonist was removed from the X-ray structure and the agonist 5′-N-ethylcarboxamidoadenosine (NECA) placed in the receptor with atomic coordinates extracted from the A_2A_AR X-ray structure (PDB: 2YDV), after aligning the two receptor transmembrane domains. GaMD simulations of the NECA-bound A_1_AR were performed in the absence and presence of two PAMs, PD81723 and VCP171. Each PAM was initially placed at least 20 Å away from the receptor (Fig. 1C). Multiple independent GaMD simulations were performed using AMBER and NAMD at different acceleration levels to investigate the PAM binding processes (Table 1).

**Table 1:** Summary of GaMD simulations performed on the adenosine A_1_ receptor (A_1_AR).

System	Method	Simulations	*∆V*_*avg*_ (kcal/mol)	*σ*_*∆V*_ (kcal/mol)	NECA Clusters	
					w/o PAM	w/ PAM
A_1_AR + NECA	GaMD_Dual (AMBER)	500 ns × 5	17.89	5.23	51	—
	GaMD_Dual (NAMD)	300 ns × 5	11.77	3.07	4	—
	GaMD_Dih (NAMD)	200 ns × 5	6.04	2.23	6	—
A_1_AR + NECA + PD81723	GaMD_Dual (AMBER)	500 ns × 5	18.36	5.29	2	2
	GaMD_Dual (NAMD)	300 ns × 5	11.14	3.07	12	9
	GaMD_Dih (NAMD)	200 ns × 5	5.65	2.14	1	1
A_1_AR + NECA + VCP171	GaMD_Dual (AMBER)	500 ns × 5	17.66	5.23	73	4

*∆V*_*avg*_ and *σ*_*∆V*_ are the average and standard deviation of the GaMD boost potential. The number of structural clusters of the orthosteric agonist NECA are calculated from the GaMD simulations in the absence or presence of PAM binding, PD81723 or VCP171, to the ECL2 allosteric site.

With AMBER, GaMD simulations boosted both the total and dihedral energetic terms (“dual-boost GaMD”)^52^ on the NECA-bound A_1_AR, NECA-bound A_1_AR in the presence of PD81723 and NECA-bound A_1_AR in the presence of VCP171 provided boost potentials of 17.89 ± 5.23 kcal/mol, 18.36 ± 5.29 kcal/mol and 17.66 ± 5.23 kcal/mol, respectively. In comparison, dual-boost GaMD simulations using NAMD showed boost potentials of 11.77 ± 3.07 kcal/mol and 11.14 ± 3.07 kcal/mol for the NECA-bound A_1_AR in the absence and presence of PD81723, respectively. Further GaMD simulations were performed by boosting the dihedrals only (“dihedral GaMD”)^53^ using NAMD. The boost potentials were 6.04 ± 2.23 kcal/mol and 5.65 ± 2.14 kcal/mol for the NECA-bound A_1_AR in the absence and presence of the PD81723, respectively (Table 1). In principle, greater average and standard deviation of the boost potential (*∆V*_*avg*_ and *σ*_*∆V*_) lead to higher acceleration in the biomolecular structural fluctuations. Therefore, the AMBER version of GaMD appeared to provide higher acceleration than the NAMD version, due to slightly different algorithms implemented for computing the potential statistics in the two packages (SI Method).

Despite the different acceleration levels, all the GaMD simulations successfully captured spontaneous binding of PD81723 and VCP171 to the A_1_AR. Traces of the diffusing PAMs obtained in GaMD simulations with PD81723 and VCP171 are shown in Figs S1 and S2, respectively. Overall, PD81723 and VCP171 bound to a pocket formed by the ECL2 with the highest probability, although the PAMs could also transiently visit other regions of the A_1_AR. This agrees with previous mutagenesis experiments that alanine substitutions of residues in the ECL2 affected binding of the PAMs^26,27^. Therefore, the GaMD simulations successfully captured spontaneous binding of the A_1_AR PAMs PD81723 and VCP171.

### GaMD predicted binding poses of PD81723 were consistent with structure-function data

**Figure 2:**
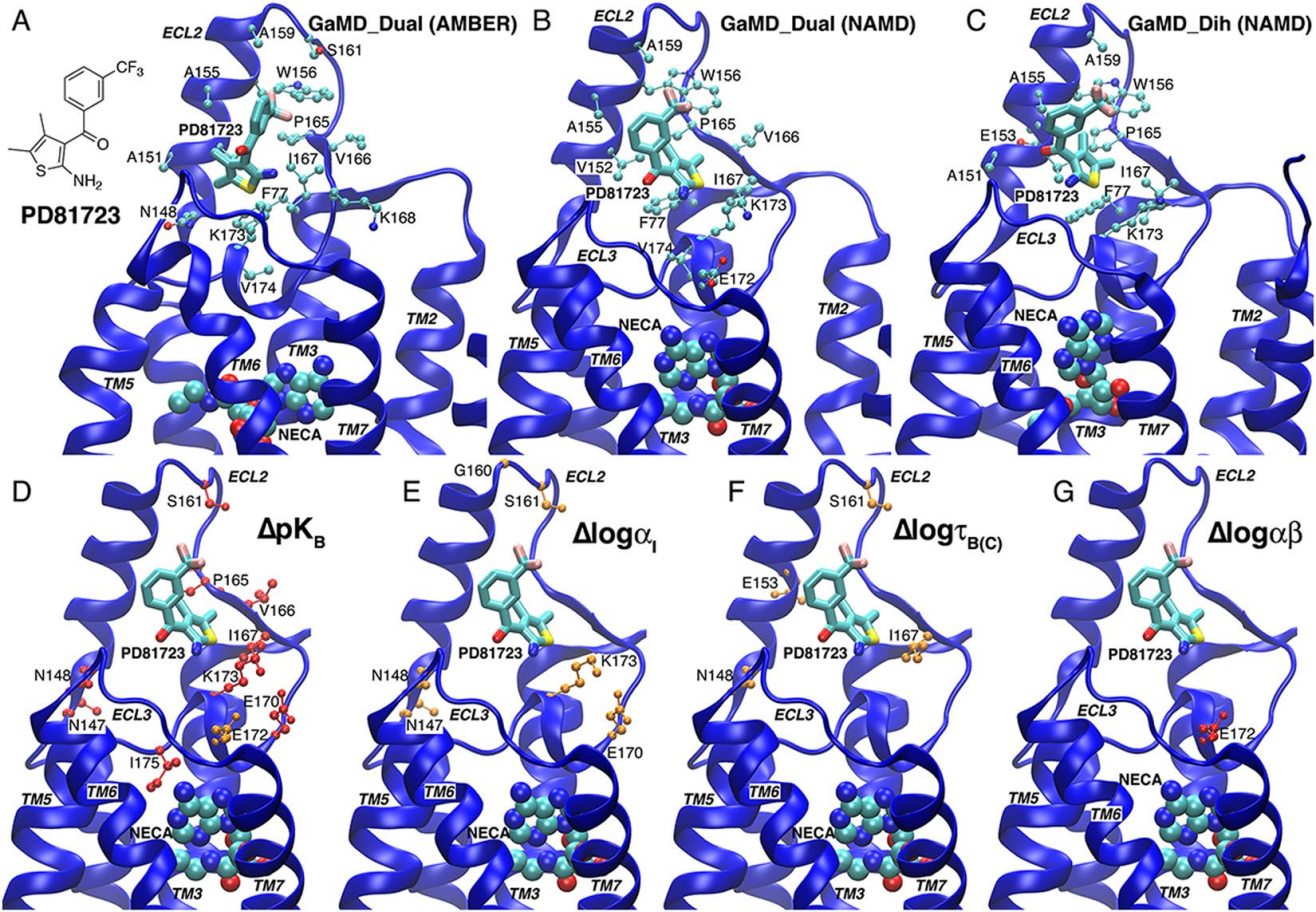
GaMD simulations predicted the A_1_AR PAM PD81723 recognized an allosteric site defined by extracellular loop 2 (ECL2): (**A**–**C**) Low-energy binding modes of PD81723 identified from (**A**) dual-boost GaMD simulations using AMBER, (**B**) dual-boost GaMD simulations using NAMD and (**C**) dihedral-boost GaMD simulations using NAMD. The receptor, orthosteric agonist NECA and PAM PD81723 are shown in ribbons, spheres and sticks, respectively. Residues found within 5 Å of the bound PD81723 are highlighted in balls-and-sticks. (**D**–**G**) A_1_AR residues for which alanine substitution were shown in a previous structure-function study^27^ to significantly decrease (orange) or enhance (red) PD81723 affinity (**D**), binding cooperativity (**E**), efficacy (**F**) or functional cooperativity (**G**).

Structural clustering of the PAMs and calculated free energies of the resulting structural clusters were performed on the GaMD simulation trajectories (see details in Methods). The lowest-energy binding poses of PD81723 at the ECL2 allosteric site obtained from dual-boost GaMD simulations using AMBER, dual-boost GaMD simulations using NAMD and dihedral GaMD simulations using NAMD are shown in Fig. 2A–C, respectively. Overall, PD81723 exhibited similar binding poses at the ECL2 allosteric site in the GaMD simulations at different acceleration levels. The trifluoro-phenyl group all pointed in the same direction towards Trp156, although the 2-amino-thiophene group was able to rotate slightly in the bottom part of the ECL2 pocket. The carbonyl oxygen always pointed towards the solvent, favoring hydrophilic interactions. The phenyl ring aligned in parallel with the short helix of ECL2, forming favorable hydrophobic interactions with protein residues Phe77, Val152, Ala155, Pro165 and Ile167. Common residues that were identified within 5 Å of the bound PD81723 in the lowest-energy poses from the GaMD simulations included Ala155, Ala159, Trp156, Pro165, Ile167, Phe77 and Lys173. Moreover, residues Asn148, Ala151, Val152, Glu153, Ser161, Val166, Lys168, Glu172 and Val174 appeared within 5 Å of PD81723 in one or two of the binding poses (Fig. 2A–C).

The lowest-energy binding poses of PD81723 obtained from the GaMD simulations were highly consistent with site-directed mutagenesis experiments^26,27^. Particularly, alanine substitution of Asn147, Asn148, Ser161, Pro165, Val166, Ile167, Glu170, Lys173 or Ile175 significantly enhanced the binding affinity (pK_B_) of PD81723, while mutation of Glu172 to alanine had the opposite effect (Fig. 2D). Alanine substitutions of residues Asn147, Asn148, Gly160, Ser161, Lys173 and Glu170 decreased the binding cooperativity (logα_I_) between PD81723 and NECA at the A_1_AR (Fig. 2E). Alanine substitutions of Asn148, Glu153, Ser161 and Ile167 decreased PAM efficacy logτ_B(C)_ (Fig. 2F). Finally, mutation of Glu172 to alanine increased the functional cooperativity (logαβ) between PD81723 and NECA for A_1_AR-mediated inhibition of cAMP accumulation (Fig. 2G).

### GaMD predicted binding mode of VCP171 was consistent with structure-function data

**Figure 3:**
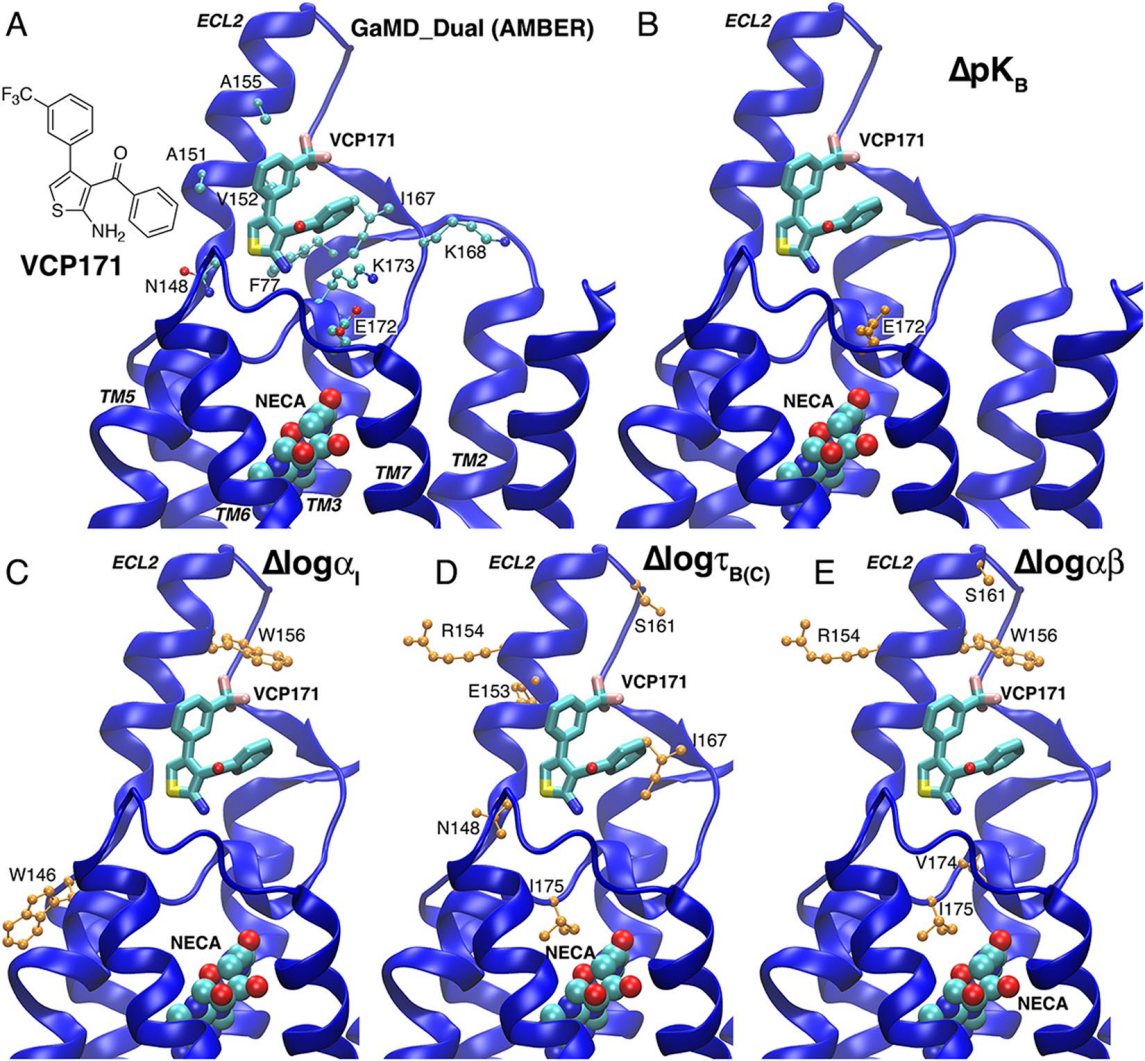
GaMD simulations predicted the A_1_AR PAM VCP171 recognized an allosteric site defined by extracellular loop 2 (ECL2): (**A**) Low-energy binding mode of VCP171 identified from dual-boost GaMD simulations using AMBER. The receptor, orthosteric agonist NECA and PAM VCP171 are shown in ribbons, spheres and sticks, respectively. Residues found within 5 Å of the bound VCP171 are highlighted in balls-and-sticks. (**B**–**E**) A_1_AR residues for which alanine substitution were shown in a previous structure-function study^27^ to significantly decrease (orange) or enhance (red) VCP171 affinity (**B**), binding cooperativity (**C**), efficacy (**D**) or functional cooperativity (**E**).

The lowest-energy binding mode of VCP171 identified from dual-boost GaMD simulations using AMBER is shown in Fig. 3A. VCP171 also recognized the putative allosteric site formed by the ECL2 and adopted a similar orientation to PD81723. The trifluoro-phenyl group was parallel to the short helix in ECL2. The thiol group pointed towards Asn148 and the additional phenyl group formed hydrophobic interactions with the Phe77 and Ile167 side chains (Fig. 3A). Residues found within 5 Å of the bound VCP171 included Phe77, Asn148, Ala151, Val152, Ala155, Ile167, Lys168, Glu172 and Lys173 (Fig. 3A). The majority of interacting residues were shared between the PAMs PD81723 and VCP171 (Fig. 2).

Residues found within 5 Å of the bound VCP171 were highly consistent with site-directed mutagenesis experiments^26,27^. Notably, mutation of Glu172 to alanine significantly decreased binding affinity of VCP171 (Fig. 3B). Alanine substitution of Trp146 and Trp156 decreased binding cooperativity between VCP171 and NECA at the A_1_AR (Fig. 3C). Alanine substitution of Asn148, Glu153, Arg154, Ser161, Ile167 and Ile175 decreased the receptor efficacy (Fig. 3D). Finally, alanine substitution of Arg154, Ser161, Trp156, Val174 and Ile175 decreased the functional cooperativity between VCP171 and NECA for A_1_AR-mediated inhibition of cAMP accumulation (Fig. 3E). Therefore, the binding mode of VCP171 obtained from the GaMD simulations was supported by experimental structure-function analysis^26,27^.

### PAM binding stabilized agonist binding within the A1AR orthosteric site

**Figure 4:**
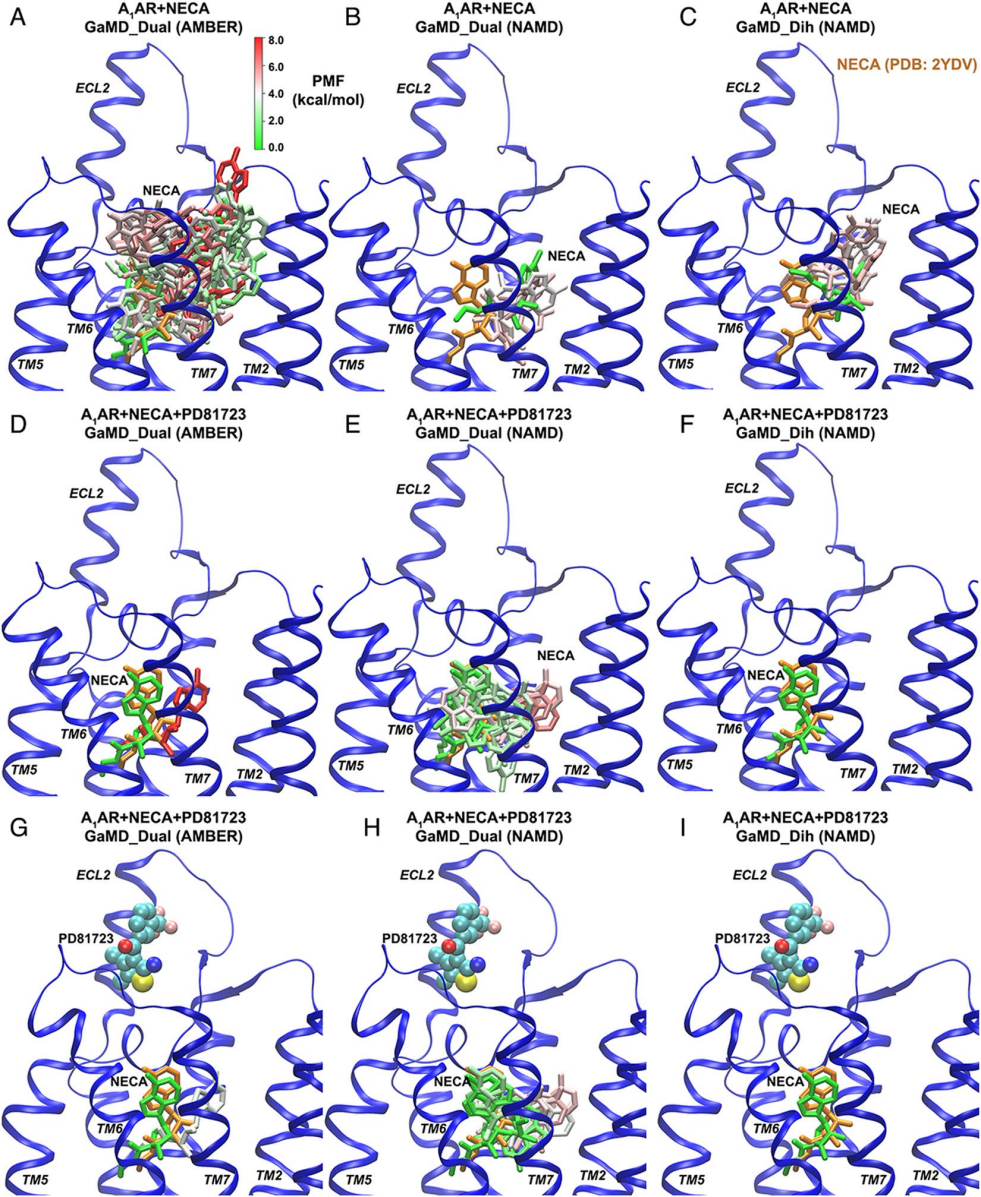
PD81723 stabilized NECA binding within the A_1_AR orthosteric site: (**A**–**C**) structural clusters of NECA identified in simulations of the “A_1_AR + NECA” system using (**A**) dual-boost GaMD with AMBER, (**B**) dual-boost GaMD with NAMD and (**C**) dihedral-boost GaMD with NAMD. (**D**–**F**) structural clusters of NECA identified in simulations of the “A_1_AR + NECA + PD81723” system with no PD81723 bound at the ECL2 allosteric site using (**D**) dual-boost GaMD with AMBER, (**E**) dual-boost GaMD with NAMD and (**F**) dihedral-boost GaMD with NAMD. (**G**–**I**) structural clusters of NECA identified in simulations of the “A_1_AR + NECA + PD81723” system with PD81723 bound at the ECL2 allosteric site using (**G**) dual-boost GaMD with AMBER, (**H**) dual-boost GaMD with NAMD and (**I**) dihedral-boost GaMD with NAMD. The receptor, orthosteric agonist (NECA) and PAM (PD81723) are shown in ribbons, sticks and spheres, respectively. NECA clusters are colored by the potential of mean force (PMF) in a green(0 kcal/mol)-white-red(8 kcal/mol) scale and the NECA conformation extracted from the 2YDV X-ray structure of the A_2A_AR with two receptor transmembrane domains aligned is shown in orange for reference.

Subsequent analysis assessed the influence of PAMs on NECA binding within the A_1_AR orthosteric site. Structural clusters of the agonist NECA were obtained in the absence and presence of PAM binding (Fig. 4). In the NECA-bound A_1_AR simulation system in the absence of an allosteric ligand, NECA sampled a large conformational space in the orthosteric pocket. Numerous structural clusters of NECA were identified from simulations of the NECA-bound A_1_AR system using dual-boost GaMD with AMBER (51 clusters, Fig. 4A), dual-boost GaMD with NAMD (4 clusters, Fig. 4B) and dihedral-boost GaMD with NAMD (6 clusters, Fig. 4C). In dual-boost GaMD simulations with AMBER, which provided the highest acceleration (Table 1), the NECA agonist was able to explore the entire orthosteric pocket (Fig. 4A). Whereas in the dual-boost and dihedral GaMD simulations using NAMD with lower acceleration levels, NECA sampled a smaller conformational space (Fig. 4B,C**)**. Accordingly, the RMSD of NECA relative to the crystal conformation obtained from the 2YDO X-ray structure of the A_2A_AR with two receptor TM domains aligned exhibited large variations during the AMBER dual-boost GaMD simulations (Fig. S3A), while the NAMD dual-boost and dihedral GaMD simulations showed smaller variations in the agonist RMSDs (Fig. S3B and S3C).

During GaMD simulations of the NECA-bound A_1_AR in the presence of PD81723, RMSD of NECA typically remained <5 Å with small variations (Fig. S4), except that it reached ~9 Å in one of the five dual-boost GaMD simulations using NAMD (“Sim5” in Fig. S4B). This suggested that PD81723 was able to stabilize NECA binding in the orthosteric site. We tracked PD81723 diffusion and identified structural clusters of NECA without and with PD81723 bound at the ECL2 allosteric site. Different numbers of structural clusters for the orthosteric agonist NECA with no PD81723 bound at the ECL2 allosteric site were obtained during the dual-boost GaMD simulations with AMBER (2 clusters), dual-boost GaMD simulations with NAMD (12 clusters) and dihedral GaMD simulations with NAMD (1 cluster) (Fig. 4D–F, Table 1). In comparison, PD81723 binding to the ECL2 allosteric site led to fewer structural clusters and smaller conformational space of NECA in the orthosteric pocket (Fig. 4 and Table 1). During the AMBER dual-boost GaMD, NAMD dual-boost GaMD and NAMD dihedral GaMD simulations, the number of structural clusters identified for NECA was 2, 9 and 1, respectively (Table 1). Upon binding of PD81723 at the ECL2 allosteric site, NECA sampled a smaller conformational space. Structural clusters of NECA identified in simulations of the “A_1_AR + NECA + PD81723” system with PD81723 bound at the ECL2 allosteric site using dual-boost GaMD with AMBER, dual-boost GaMD with NAMD and dihedral-boost GaMD with NAMD are shown in Fig. 4G–I. Movement of the NECA agonist was greatly reduced in the presence of PD81723. Therefore, the PD81723 PAM stabilized agonist binding at the orthosteric site of the A_1_AR.

### Agonist dissociation was observed in the absence of PAM binding

**Figure 5:**
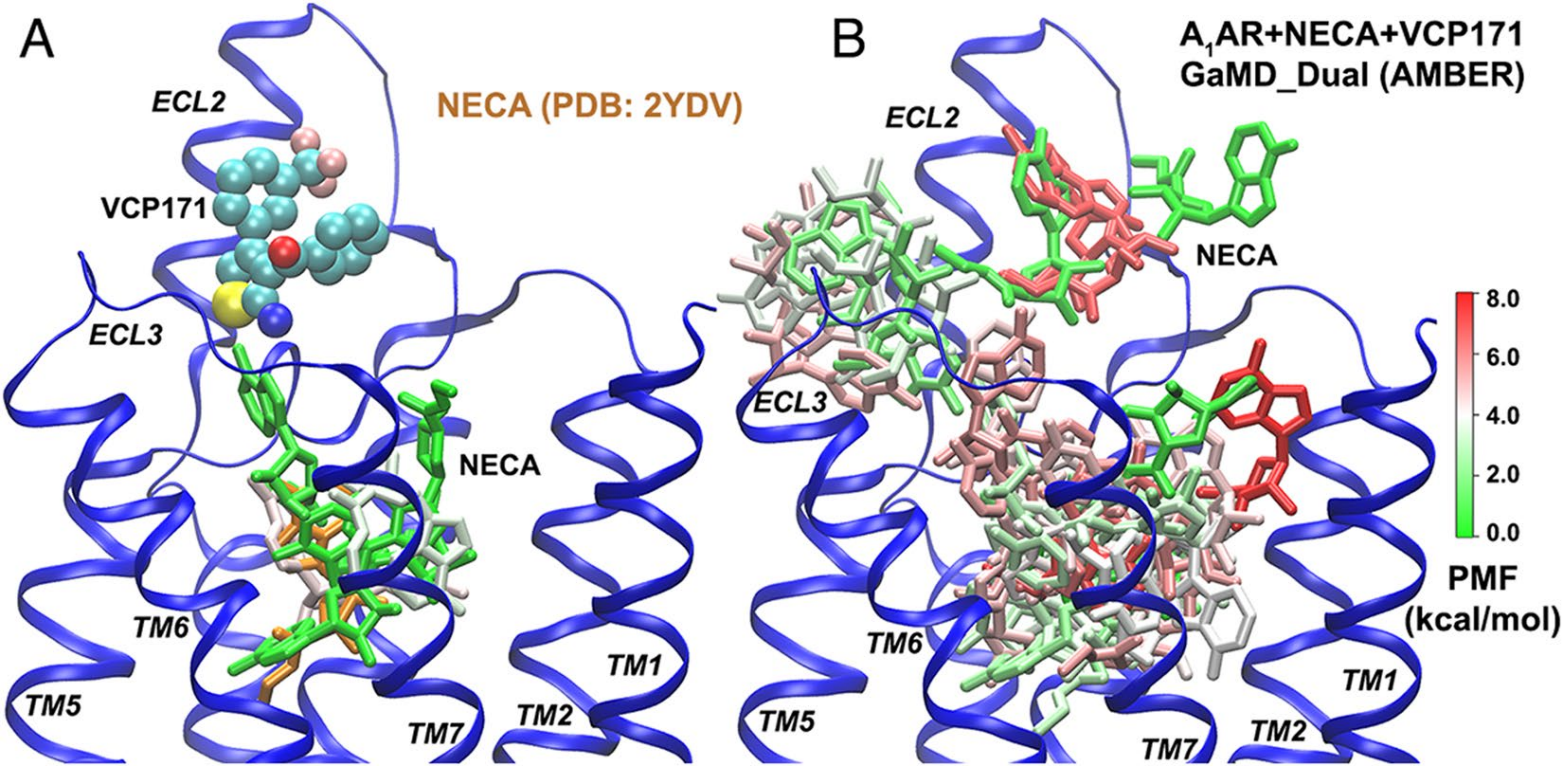
Structural clusters of NECA identified in dual-boost GaMD simulations using AMBER of the “A_1_AR + NECA + VCP171” system: (**A**) binding of NECA in the A_1_AR orthosteric site was stabilized upon VCP171 binding to the ECL2 allosteric site. (**B**) Dissociation of NECA was observed in the absence of VCP171 binding to the ECL2 allosteric site. The receptor, orthosteric agonist (NECA) and PAM (VCP171) are shown in ribbons, sticks and spheres, respectively. NECA clusters are colored by free energy in a green(0 kcal/mol)-white-red(8 kcal/mol) scale and the NECA conformation extracted from the 2YDV X-ray structure of the A_2A_AR with two receptor transmembrane domains aligned is shown in orange for reference.

Dual-boost GaMD simulations using AMBER were also performed on the system of NECA-bound A_1_AR in the presence of the VCP171 PAM. While NECA showed small movements with mostly <10 Å RMSD relative to the 2YDV crystal conformation (Fig. S5), it escaped out of the receptor with >20 Å RMSD in one of the five GaMD simulations (“Sim3”). The structural clusters of NECA were identified from the GaMD simulations with and without VCP171 bound at the ECL2 allosteric site (Fig. 5). VCP171 binding to ECL2 greatly limited the conformational space of NECA agonist with only four structural clusters identified in the receptor orthosteric pocket (Fig. 5A and Table 1).

In the absence of VCP171 binding to the ECL2 allosteric site, NECA sampled a significantly larger conformational space and even dissociated from the A_1_AR (Fig. 5B). A large number of structural clusters (73 clusters) were identified for the diffusing NECA (Table 1). The lowest energy clusters depict an agonist dissociation pathway, which connect the receptor orthosteric site, extracellular opening between the ECL2/ECL3, the allosteric site formed by the ECL2 and finally the solvent (Fig. 5B). This is consistent with previous simulation findings that ligand binding through the ECL2/ECL3 opening is an energetically preferred pathway of class A GPCRs^34,49,58,59^. Furthermore, the open pocket formed by only the ECL2 serves as an additional metastable binding site of the agonist, as well as the target site of PAMs in the A_1_AR.

### PAM binding promotes the formation of a salt bridge E172^ECL2^-K265^ECL3^ in the A_1_AR extracellular vestibule

**Figure 6:**
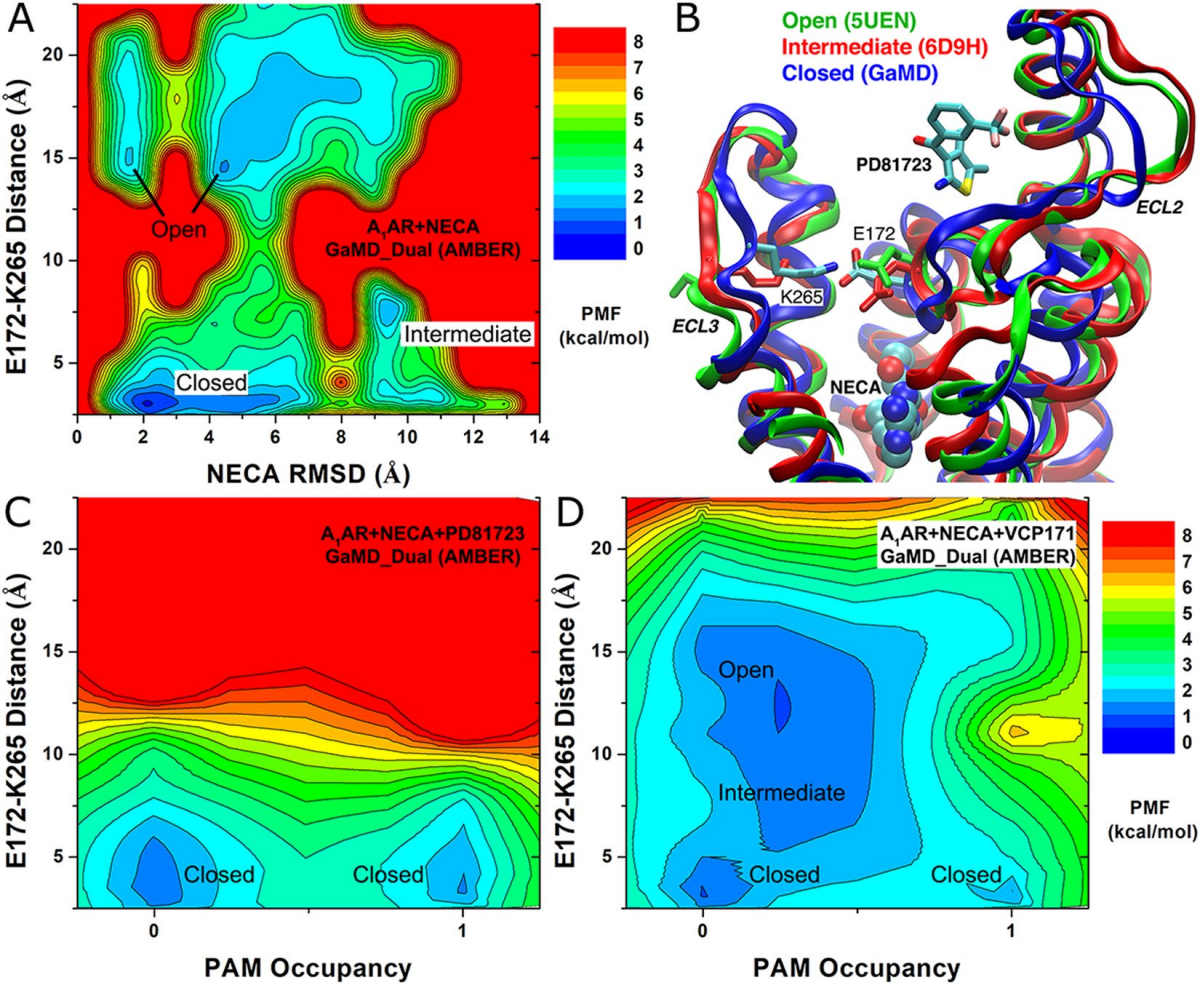
PAM binding closed a salt bridge E172^ECL2^-K265^ECL3^ in the A_1_AR extracellular vestibule: (**A**) A 2D PMF profile of the E172^ECL2^-K265^ECL3^ distance and NECA RMSD relative to the starting bound conformation obtained from AMBER dual-boost GaMD simulation of the “A_1_AR + NECA” system. The C_δ_ atom in E172 and N_ζ_ atom in K265 were used to calculate the distance. (**B**) Three low-energy states, “Open”, “Intermediate” and “Closed”, identified in (**A**) are shown using the X-ray structure of antagonist DU172-bound A_1_AR (PDB: 5UEN), cryo-EM structure of adenosine-G_i_-bound A_1_AR (PDB: 6D9H) and GaMD predicted structure of the NECA and PAM PD81723 co-bound A_1_AR. (**C**,**D**) 2D PMF profiles of the E172^ECL2^-K265^ECL3^ distance and PAM occupancy at the ECL2 allosteric site obtained from AMBER dual-boost GaMD simulations of the (**C**) “A_1_AR + NECA + PD81723” and (**D**) “A_1_AR + NECA + VCP171” systems. PAM binding biased conformation ensemble of the E172^ECL2^-K265^ECL3^ salt bridge towards the closed state, leading to stabilized agonist binding at the orthosteric site.

We have examined protein residue interactions in the A_1_AR extracellular domains to understand the allosteric mechanism of stabilized agonist interactions within the orthosteric site in the presence of PAM binding to ECL2. Simulation analysis revealed a salt bridge between Glu172^ECL2^-Lys265^ECL3^ in the extracellular mouth of the A_1_AR. In particular, the favorable hydrophobic interactions between the PAM and ECL2 within the allosteric pocket positioned Glu172^ECL2^ to extend its side chain towards residue Lys265^ECL3^. The Glu172^ECL2^-Lys265^ECL3^ salt bridge in the A_1_AR extracellular mouth was then closed upon PAM binding to the ECL2 allosteric site (Fig. 6). This predicted interaction is consistent with mutation experimental data that substitution of Glu172 to alanine significantly affected binding affinity of PD81723 and functional cooperativity between the PAM and agonist^26,27^ (Fig. 2).

A 2D PMF profile of the Glu172^ECL2^-Lys265^ECL3^ distance and RMSD of the NECA agonist relative to the starting bound conformation was calculated from AMBER dual-boost GaMD simulations of the NECA-bound A_1_AR system (Fig. 6A**)**. Three low-energy conformational states, “open”, “intermediate” and “closed”, were identified from the free energy profile. Notably, this salt bridge adopted the open conformation in the X-ray structure of antagonist DU172-bound A_1_AR (PDB: 5UEN)^61^ and intermediate conformation in the cryo-EM structure of adenosine-G_i_-bound A_1_AR (PDB: 6D9H)^62^, for which the Glu172^ECL2^-Lys265^ECL3^ distance is 14.82 Å/15.14 Å (dimer in the 5UEN structure) and 7.12 Å, respectively. Upon PAM binding to the A_1_AR, the salt bridge changed to the closed conformation with 3.0 Å distance between the Glu172^ECL2^ and Lys265^ECL3^ (Fig. 6B). In addition, 2D PMF profiles of the Glu172^ECL2^-Lys265^ECL3^ distance and the occupancy of PAMs at the ECL2 allosteric site were calculated from AMBER dual-boost GaMD simulations of the “A_1_AR + NECA + PD81723” and “A_1_AR + NECA + VCP171” systems (Fig. 6C,D). Time courses of the PAM occupancy showed that PD81723 bound typically fast (within ~30 ns) to the ECL2 allosteric site in all the five GaMD simulations (Fig. S6A). Accordingly, the E172^ECL2^-K265^ECL3^ distance decreased to ~3 Å (Fig. S7B) and the salt bridge stayed mostly closed in GaMD simulations of the “A_1_AR + NECA + PD81723” system (Fig. 6C). In comparison, VCP171 rarely bound to the ECL2 allosteric site during two of the five GaMD simulations (Sim3 and Sim4 as shown in Fig. S6B). The salt bridge sampled closed, intermediate and open conformations in the “A_1_AR + NECA + VCP171” system without VCP171 binding to the ECL2, but it was confined to the closed state upon binding of VCP171 to the ECL2 (Figs 6D and S7C). Therefore, PAM binding to the ECL2 allosteric site biased conformational ensemble of the Glu172^ECL2^-Lys265^ECL3^ salt bridge towards the closed state, leading to stabilized agonist binding at the orthosteric site. This provided important insights into the mechanism of allosteric modulation in the A_1_AR.

## Discussion

In this study, we have determined structural binding modes of prototypical PAMs in the A_1_AR through extensive GaMD enhanced simulations. The GaMD simulations have been performed using the AMBER and NAMD simulation packages at different acceleration levels. In the GaMD simulations, the A_1_AR PAMs bound to an allosteric site formed by ECL2, a finding that was highly consistent with experimental data^26,27^. Many of the A_1_AR residues identified within 5 Å of bound PAMs in the GaMD simulations, including ECL2 residues Asn148, Glu153, Ser161, Ile167 and Glu172, have previously been suggested to be important for PAM binding in structure-function studies. These studies demonstrated that alanine substitution of these residues significantly influenced PAM affinity, cooperativity and/or efficacy^26,27^. Therefore, these findings suggest the A_1_AR PAM allosteric site resides within the extracellular vestibule, predominantly involving interactions with ECL2.

Furthermore, the GaMD simulations showed that the PAMs stabilized agonist binding at the A_1_AR orthosteric site. Specifically, compared to simulations performed in the absence of an allosteric ligand, agonist movement within the orthosteric site decreased in the presence of a PAM bound to the ECL2 allosteric site. This was correlated with conformational change in the Glu172^ECL2^-Lys265^ECL3^ distance in the A_1_AR extracellular mouth. In GaMD simulations of the NECA-bound A_1_AR system, the Glu172^ECL2^-Lys265^ECL3^ salt bridge sampled three low-energy conformational states (“Open”, “Intermediate” and “Closed”). Importantly, the open and intermediate conformations have been determined in the X-ray structure of antagonist DU172-bound A_1_AR (PDB: 5UEN)^61^ and the cryo-EM structure of adenosine-G_i_-bound A_1_AR (PDB: 6D9H)^62^, respectively. New high resolution A_1_AR structures co-bound with a PAM and agonist are required to confirm the predicted PAM-mediated stabilization of the Glu172^ECL2^-Lys265^ECL3^ salt bridge in the closed conformation. Moreover, further GaMD simulations on PAM binding to the active A_1_AR using the recent cryo-EM structure of the adenosine-bound A_1_AR–Gi complex are subject to future study. Nevertheless, the present GaMD simulations have provided important insights into the mechanism of allosteric modulation at the A_1_AR.

In the absence of PAM binding to ECL2, the orthosteric agonist explores a significantly larger conformational space and could even dissociate from the A_1_AR through an opening between ECL2 and ECL3. This pathway connecting the orthosteric site and ECL2/ECL3 opening has also been suggested as an energetically preferred ligand binding pathway of other class A GPCRs, including the β_2_AR^34^, M_2_^59^ and M_3_ mAChRs^53^. An orthosteric antagonist ZM241385 has also been observed to dissociate from the A_2A_AR through a similar pathway in previous Anton simulations using TAMD^49^.

The extracellular allosteric site formed by only the ECL2 appears to be unique in the A_1_AR. Such an allosteric target site has not been identified in other GPCRs so far^63^. In the M_2_ receptor, a PAM LY2119620 binds to the receptor extracellular vestibule formed by the TM2, TM6 and TM7 in addition to ECL2 as identified in X-ray crystallography^36^ and MD simulations^35^. Sequence alignment of the four subtypes of ARs showed that while the seven TM helix bundle of the A_1_AR shares high similarity with the A_2A_AR (71%), A_2B_AR (70%) and A_3_AR (77%), the similarity is significantly reduced in the ECLs, being 43% for A_2A_AR, 45% for A_2B_AR and 35% for A_3_AR when compared with the A_1_AR. The entire ECL2 has low sequence conservation among the ARs (Fig. S8A). Comparison of X-ray structures of the A_1_AR (PDB: 5UEN)^60^ with the adenosine-bound A_2A_AR (PDB: 2YDO)^64^ also showed significant differences in the ECL2 conformations. The ECL2 forms a longer helix in the A_1_AR than in the A_2A_AR and the helix adopts distinct orientations in the two receptors^60^. Two disulfide bonds, SSB1 and SSB2 that anchor ECL2 to ECL1 and Cys^3.22^ in A_2A_AR, respectively, are not conserved in the A_1_AR (Fig. S8B). This likely leads to higher flexibility of ECL2 in the A_1_AR and could play an important role in the binding of selective PAMs.

In summary, we have successfully identified a structural binding mode of A_1_AR PAMs through extensive all-atom GaMD simulations that is consistent with previous experimental structure-function analysis. The GaMD simulations provide important insights into the allosteric modulation mechanism of the A_1_AR, predicting that a salt bridge between Glu172^ECL2^-Lys265^ECL3^ in the A_1_AR extracellular mouth is closed upon PAM binding to the ECL2, leading to stabilized agonist binding within the orthosteric site. The ECL2 appears to serve as the target site for A_1_AR PAMs, as well as an additional metastable binding site for orthosteric agonists. With remarkable divergence of residue sequences and conformations, the ECL2 presents an exciting target site for designing selective allosteric drugs of the A_1_AR. The GaMD simulations, together with mutagenesis data, will greatly facilitate future structure-based computer-aided drug design of novel A_1_AR PAMs.

## Methods

### Gaussian Accelerated Molecular Dynamics

Gaussian accelerated molecular dynamics (GaMD) is an enhanced sampling technique that works by adding a harmonic boost potential to reduce the system energy barriers^52^. GaMD accelerates biomolecular simulations by orders of magnitude. GaMD does not require predefined collective variables. Compared with the enhanced sampling methods that rely on careful selection of the collective variables, GaMD is of particular advantage for studying “free” protein-ligand binding processes^37,52^. Moreover, because the boost potential follows a Gaussian distribution, biomolecular free energy profiles can be properly recovered through cumulant expansion to the second order^52^. GaMD thus solves the energetic reweighting problem as encountered in the previous aMD method^50,65^ for free energy calculations. GaMD has been implemented in the widely used AMBER^52,66^ and NAMD^53^ packages. It has allowed us to characterize protein folding, protein-ligand binding, protein-protein binding and protein-nucleic acid interactions^52,53,59,67,68^. Details of the method have been described in previous studies^52,53^. A brief summary is provided here.

Consider a system with *N* atoms at positions $\mathop{r}\limits^{\rightharpoonup }$ = {$\mathop{r}\limits^{\rightharpoonup }$_1_ …,$\mathop{r}\limits^{\rightharpoonup }$_*N*_}. When the system potential *V*($\mathop{r}\limits^{\rightharpoonup }$) is lower than a reference energy *E*, the modified potential V^*^($\mathop{r}\limits^{\rightharpoonup }$) of the system is calculated as:

1 $$\begin{array}{c}\,\,\,{V}^{\ast }(\mathop{r}\limits^{\rightharpoonup })=V(\mathop{r}\limits^{\rightharpoonup })+{\rm{\Delta }}V(\mathop{r}\limits^{\rightharpoonup }),\\ {\rm{\Delta }}V(\mathop{r}\limits^{\rightharpoonup })=\{\begin{array}{rr}\frac{1}{2}k{(E-V(\mathop{r}\limits^{\rightharpoonup }))}^{2}, & V(\mathop{r}\limits^{\rightharpoonup }) < E\\ 0, & V(\mathop{r}\limits^{\rightharpoonup })\ge E\end{array}\end{array}$$

where *k* is the harmonic force constant. The two adjustable parameters *E* and *k* are automatically determined based on three enhanced sampling principles^52^. The reference energy needs to be set in the following range:

2 $${V}_{max}\le E\le {V}_{min}+\frac{1}{k},$$

where *V*_*max*_ and *V*_*min*_ are the system minimum and maximum potential energies. To ensure that Eqn. (2) is valid, *k* must satisfy: $k\le \frac{1}{{V}_{max}-{V}_{min}}$. Let us define $k\equiv {k}_{0}\frac{1}{{V}_{max}-{V}_{min}}$, then 0 < *k*_0_ ≤ 1. The standard deviation of Δ*V* needs to be small enough (i.e., narrow distribution) to ensure proper energetic reweighting^69^: ${\sigma }_{\Delta V}=k(E-{V}_{avg}){\sigma }_{V}\le {\sigma }_{0}$ where *V*_*avg*_ and *σ*_*V*_ are the average and standard deviation of the system potential energies, *σ*_Δ*V*_ is the standard deviation of Δ*V* with *σ*_0_ as a user-specified upper limit (e.g., 10 *k*_*B*_T) for proper reweighting. When *E* is set to the lower bound *E* = *V*_*max*_, *k*_0_ can be calculated as:

3 $${k}_{0}=\,{\rm{\min }}\,(1.0,\,{k^{\prime} }_{0})=\,{\rm{\min }}\,(1.0,\,\frac{{\sigma }_{0}}{{\sigma }_{V}}\frac{{V}_{max}-{V}_{min}}{{V}_{max}-{V}_{avg}}).$$

Alternatively, when the threshold energy *E* is set to its upper bound $E={V}_{min}+\frac{1}{k}$, *k*_0_ is set to:

4 $${k}_{0}={k^{\prime\prime} }_{0}\equiv (1-\frac{{\sigma }_{0}}{{\sigma }_{V}})\frac{{V}_{max}-{V}_{min}}{{V}_{max}-{V}_{avg}},$$

if ${k^{\prime\prime} }_{0}$ is found to be between 0 and 1. Otherwise, *k*_0_ is calculated using Eqn. (3).

Similar to aMD, GaMD provides options to add only the total potential boost Δ*V*_*P*_, only dihedral potential boost Δ*V*_*D*_, or the dual potential boost (both Δ*V*_*P*_ and Δ*V*_*D*_). The dual-boost simulation generally provides higher acceleration than the other two types of simulations for enhanced sampling^51^. The simulation parameters comprise of the threshold energy values and the effective harmonic force constants, *k*_0*P*_ and *k*_0*D*_ for the total and dihedral potential boost, respectively.

For energetic reweighting of GaMD simulations to calculate potential of mean force (PMF), the probability distribution along a reaction coordinate is written as p^*^(*A*). Given the boost potential ${\rm{\Delta }}V(\mathop{r}\limits^{\rightharpoonup })$ of each frame, p^*^(*A*) can be reweighted to recover the canonical ensemble distribution, *p*(*A*), as:

5 $$p({A}_{j})={p}^{\ast }({A}_{j})\,\frac{{\langle {e}^{\beta {\rm{\Delta }}V(\mathop{r}\limits^{\rightharpoonup })}\rangle }_{j}}{{\sum }_{i=1}^{M}\,{\langle {p}^{\ast }({A}_{i}){e}^{\beta {\rm{\Delta }}V(\mathop{r}\limits^{\rightharpoonup })}\rangle }_{i}},\,j=1,\ldots ,\,M,$$

where *M* is the number of bins, *β* = *k*_*B*_*T* and $\langle {e}^{\beta {\rm{\Delta }}V(\mathop{r}\limits^{\rightharpoonup })}{\rangle }_{j}$ is the ensemble-averaged Boltzmann factor of ${\rm{\Delta }}V(\mathop{r}\limits^{\rightharpoonup })$ for simulation frames found in the *j*^th^ bin. The ensemble-averaged reweighting factor can be approximated using cumulant expansion:

6 $$\langle {e}^{\beta {\rm{\Delta }}V(\mathop{r}\limits^{\rightharpoonup })}\rangle =exp\,\{\sum _{k=1}^{\infty }\frac{{\beta }^{k}}{k!}{C}_{k}\},$$

where the first two cumulants are given by:

7 $$\begin{array}{c}{C}_{1}=\langle {\rm{\Delta }}V\rangle ,\\ {C}_{2}=\langle {\rm{\Delta }}{V}^{2}\rangle -{\langle {\rm{\Delta }}V\rangle }^{2}={\sigma }_{v}^{2}.\end{array}$$

The boost potential obtained from GaMD simulations usually follows near-Gaussian distribution^68^. Cumulant expansion to the second order thus provides a good approximation for computing the reweighting factor^52,69^. The reweighted free energy *F*(*A*) = −*k*_*B*_*T* ln *p*(*A*) is calculated as:

8 $$F(A)={F}^{\ast }(A)-\sum _{k=1}^{2}\,\frac{{\beta }^{k}}{k!}{C}_{k}+{F}_{c},$$

where F^*^(*A*) = −*k*_*B*_*T* ln p^*^(*A*) is the modified free energy obtained from GaMD simulation and *F*_*c*_ is a constant.

### System Setup

The first X-ray crystal structure of the A_1_AR (PDB: 5UEN)^60^ was used to set up the simulation system. After removal of antagonist, the NECA agonist was placed in the orthosteric pocket with atomic coordinates extracted from the A_2A_AR X-ray structure (PDB: 2YDV) after aligning the two receptor transmembrane domains. Four molecules of each PAM (PD81723 and VCP171) were initially placed >20 Å away from the receptor.

Two systems “A_1_AR + NECA + PD81723” and “A_1_AR + NECA + VCP171” were prepared for the simulations. In addition, the system NECA-bound A_1_AR in the absence of PAMs was also added for comparison (Table 1) The protein that was fused into the receptor to replace intracellular loop 3 (ICL3) for crystallizing the receptor structure was omitted. All chain termini were capped with neutral groups (acetyl and methylamide). The disulphide bonds that were resolved in the crystal structure were maintained in the simulations. Using the *psfgen* plugin in VMD^70^, protein residues were set to the standard CHARMM protonation states at neutral pH. Then the receptor was inserted into a palmitoyl-oleoyl-phosphatidyl-choline (POPC) bilayer with all overlapping lipid molecules removed using the *Membrane* plugin in VMD^70^. The system charges were then neutralized at 0.15 M NaCl using the *Solvate* plugin in VMD^70^. The simulation systems of the A_1_AR initially measured about 97 × 85 × 106 Å^3^ with 152 lipid molecules, ~15,600 water molecules and a total of ~72,300 atoms. Periodic boundary conditions were applied on the simulation systems.

### Simulation Protocols

The CHARMM36 parameter set^71^ was used for the protein and POPC lipids. For agonist NECA and PAMs PD81723 and VCP171, the force field parameters were obtained from the CHARMM ParamChem web server^27,72^. Initial energy minimization and thermalization of the A_1_AR system follow the same protocol as used in the previous GPCR simulations^58^. In the present GaMD simulation, the threshold energy *E* for adding boost potential is set to the lower bound^52,53^. The simulations included 2 ns cMD, 50 ns equilibration after adding the boost potential and then multiple independent production runs lasting 200–500 *ns* with randomized atomic velocities. The GaMD simulations are summarized in Table 1.

For the “A_1_AR + NECA” and “A_1_AR + NECA + PD81723” systems, GaMD simulations were performed using AMBER at the dual-boost level^52^, and NAMD at the dual-boost and dihedral acceleration levels^53^. For the “A_1_AR + NECA + VCP171” system, dual-boost GaMD simulations using AMBER were performed. The GaMD simulations were carried out using AMBER 16^52,73^ and/or NAMD2.13^53,74^. GaMD production frames were saved every 0.1 ps for analysis.

### Simulation Analysis

The VMD^70^ and CPPTRAJ^75^ tools were used for trajectory analysis. The Density Based Spatial Clustering of Applications with Noise (DBSCAN) algorithm^76^ was applied to cluster the diffusing ligand molecules for identifying their highly populated binding conformations. The frames were sieved at a stride of 200 for clustering. The remaining frames were assigned to the closest cluster afterwards. The distance cutoff for DBSCAN clustering was set to 4 Å for the PAMs and 1.5 Å for the NECA agonist. Finally, the *PyReweighting* toolkit^69^ was applied to compute free energy values of the ligand structural clusters.

## Electronic supplementary material

Supplementary Information
