# Supplementary material for: Structural Basis for Binding of Allosteric Drug Leads in the Adenosine A1 Receptor
Source: Sci Rep. 2018 Nov 15;8:16836. doi: 10.1038/s41598-018-35266-x (PMC6237911; doi:10.1038/s41598-018-35266-x)
Supplement: Supplementary file 1 — Supplementary Information [file 41598_2018_35266_MOESM1_ESM.docx]

**Supplementary Information**

for “Structural Basis for Binding of Allosteric Drug Leads in the Adenosine A_1_ Receptor” by Yinglong Miao, Apurba Bhattarai, Anh TN Nguyen, Arthur Christopoulos and Lauren T. May.

# Method

## AMBER and NAMD versions of GaMD

In AMBER, the average and standard deviation of potential energies were computed with a running time window (500 *ps* for the A_1_AR simulations)^1^. In NAMD, they were calculated by collecting the potential values since beginning of the simulation, excluding the cMD and GaMD preparation steps^2^. This implementation difference in AMBER and NAMD generated different boost potentials in GaMD simulations of the A_1_AR as listed in **Table 1**. Due to the different algorithms implemented for computing the potential statistics in AMBER and NAMD, the AMBER version appears to generate higher boost potential than the NAMD version. Nevertheless, the presented GaMD simulations with AMBER and NAMD all consistently captured spontaneous binding of PAMs to the A_1_AR.

**References:**

1 Miao, Y., Feher, V. A. & McCammon, J. A. Gaussian Accelerated Molecular Dynamics: Unconstrained Enhanced Sampling and Free Energy Calculation. *J Chem Theory Comput* **11**, 3584-3595, doi:10.1021/acs.jctc.5b00436 (2015).

2 Pang, Y. T., Miao, Y., Wang, Y. & McCammon, J. A. Gaussian Accelerated Molecular Dynamics in NAMD. *J Chem Theory Comput* **13**, 9-19, doi:10.1021/acs.jctc.6b00931 (2017).


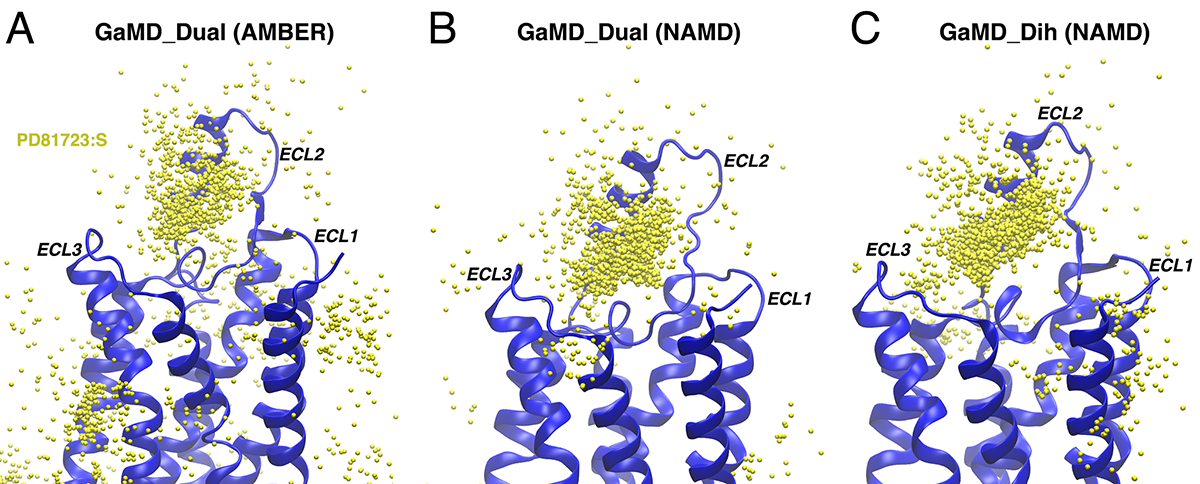


**Fig. S1** Traces of the PD81723 PAM (yellow beads shown for the ligand sulfur atom) observed in (A) dual-boost GaMD simulations using AMBER, (B) dual-boost GaMD simulations using NAMD and (C) dihedral-boost GaMD simulations using NAMD of the A_1_AR (blue ribbons).


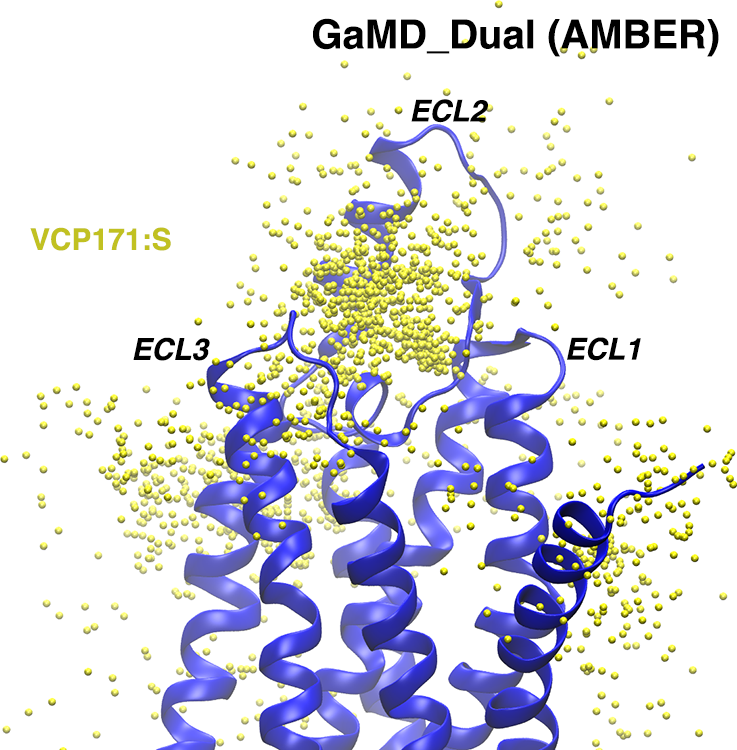


**Fig. S2** Traces of the VCP171 PAM (yellow beads shown for the ligand sulfur atom) observed in dual-boost GaMD simulations using AMBER of the A_1_AR (blue ribbons).


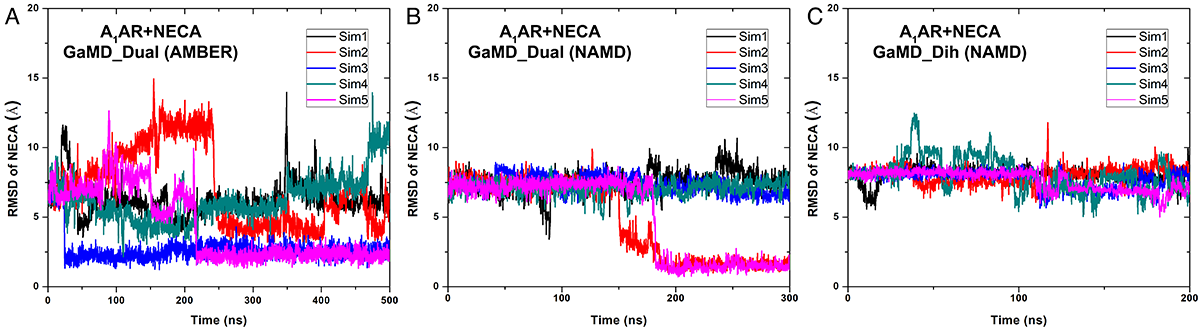


**Fig. S3** Time courses of RMSDs of the NECA agonist between simulation frames of the “A_1_AR+NECA” system and the reference crystal conformation obtained from the A_2A_AR-NECA X-ray structure (PDB: 2YDV) after aligning the two receptor transmembrane domains in the (A) dual-boost GaMD simulations using AMBER, (B) dual-boost GaMD simulations using NAMD and (C) dihedral-boost GaMD simulations using NAMD.


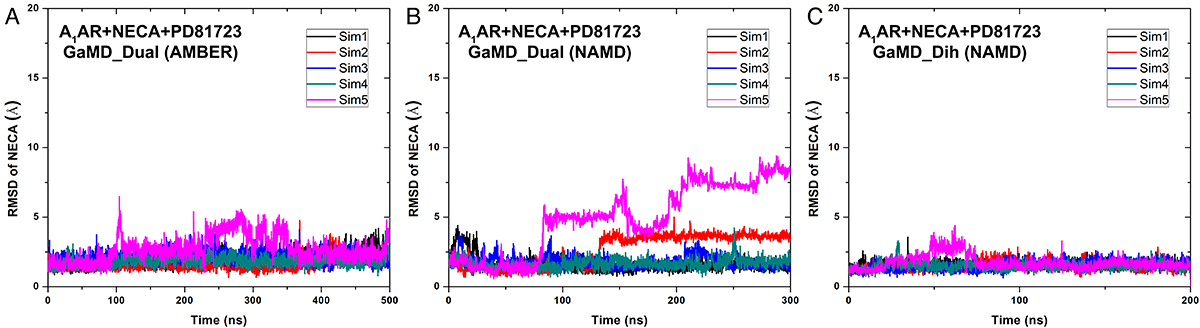


**Fig. S4** Time courses of RMSDs of the NECA agonist between simulation frames of the “A_1_AR+NECA+PD81723” system and the reference crystal conformation obtained from the A_2A_AR-NECA X-ray structure (PDB: 2YDV) after aligning the two receptor transmembrane domains in the (A) dual-boost GaMD simulations using AMBER, (B) dual-boost GaMD simulations using NAMD and (C) dihedral-boost GaMD simulations using NAMD.


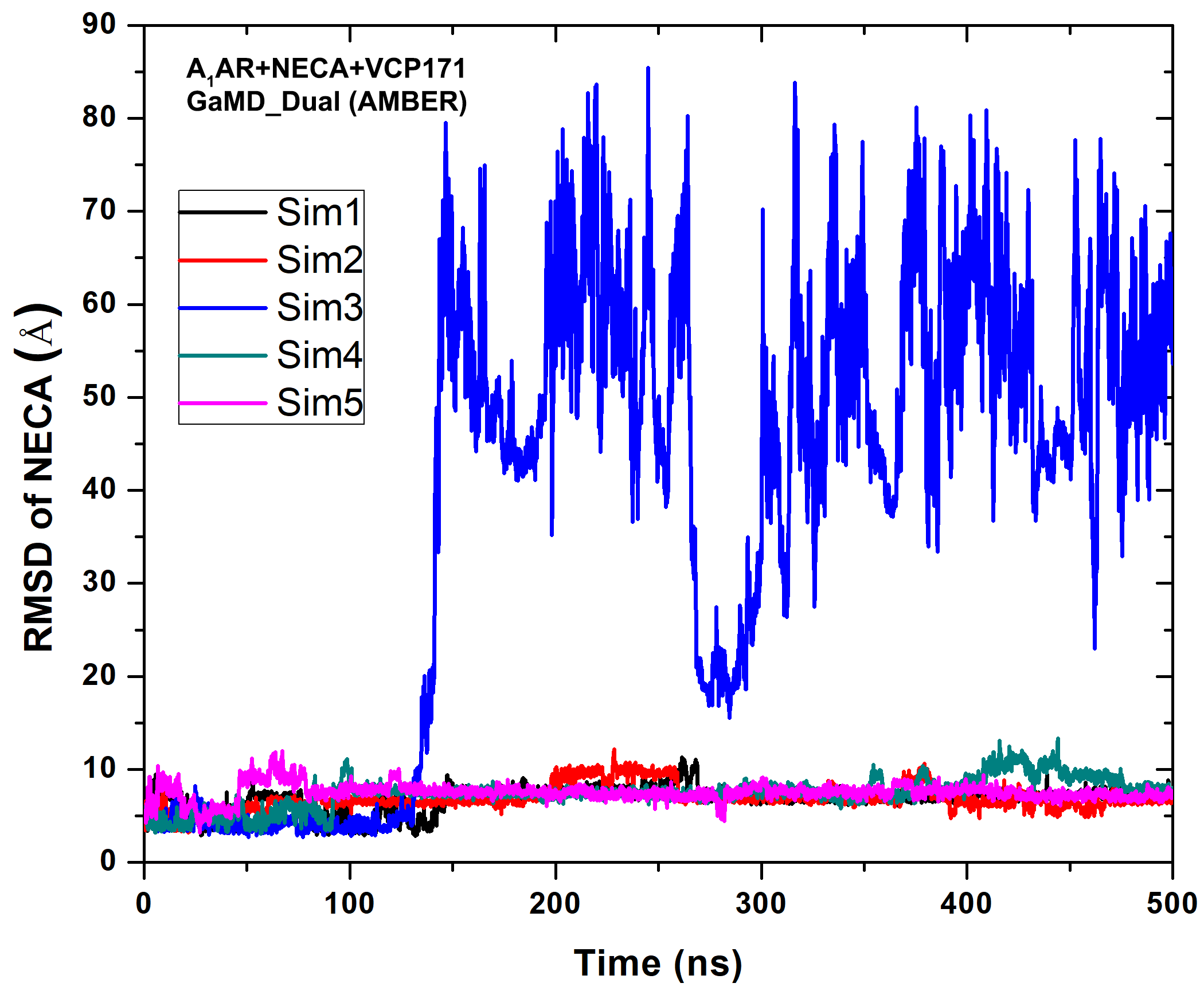


**Fig. S5** Time courses of RMSDs of the NECA agonist between simulation frames of the “A_1_AR+NECA+VCP171” system and the reference crystal conformation obtained from the A_2A_AR-NECA X-ray structure (PDB: 2YDV) after aligning the two receptor transmembrane domains in the dual-boost GaMD simulations using AMBER.


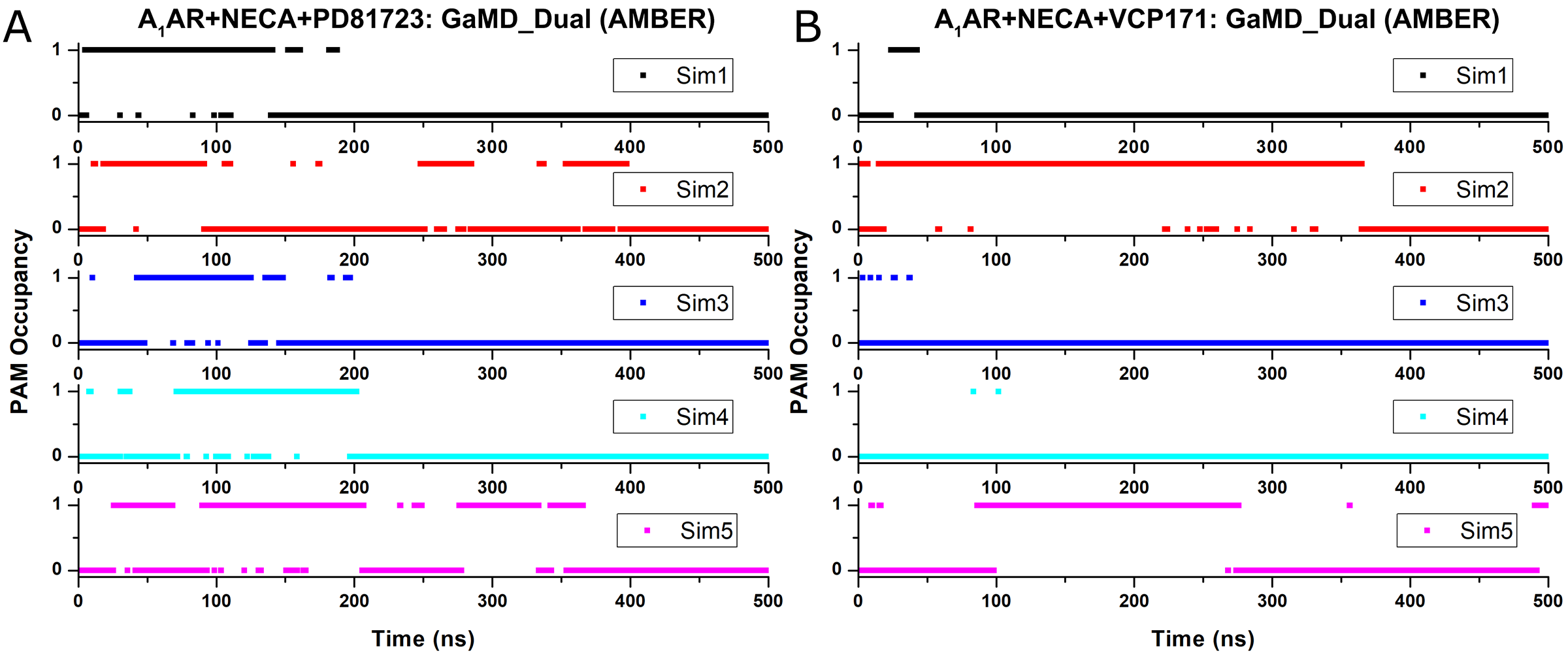


**Fig. S6** Time courses of the PAM occupancy obtained from dual-boost GaMD simulations using AMBER on the (A) “A_1_AR+NECA+PD81723” and (B) “A_1_AR+NECA+VCP171” systems.


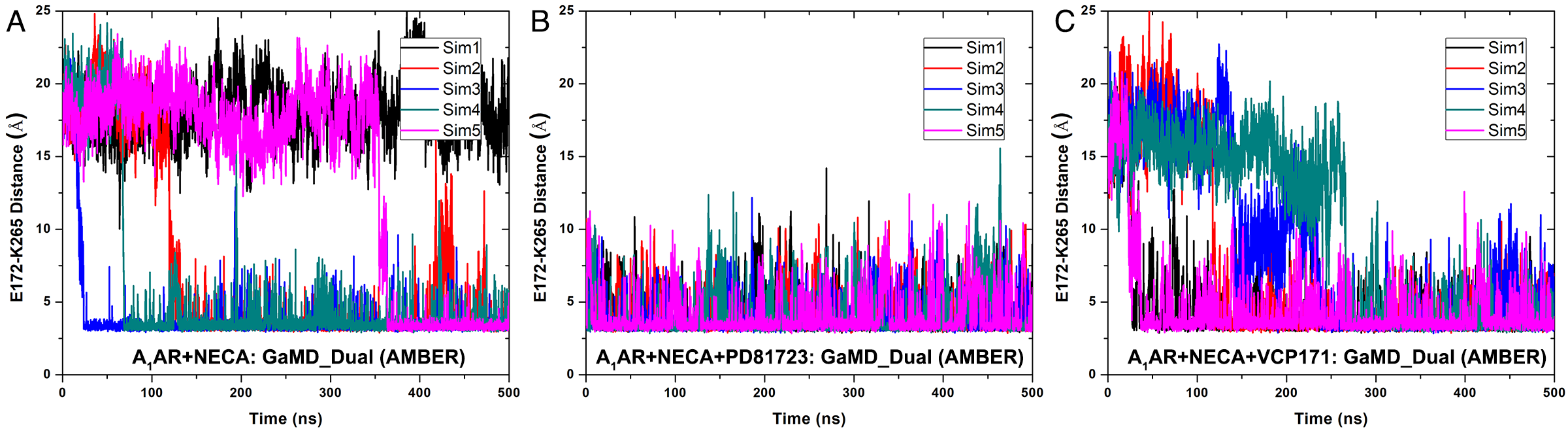


**Fig. S7** Time courses of the E172^ECL2^-K265^ECL3^ distance obtained from dual-boost GaMD simulations using AMBER on the (A) “A_1_AR+NECA”, (B) “A_1_AR+NECA+PD81723” and (C) “A_1_AR+NECA+VCP171” systems.


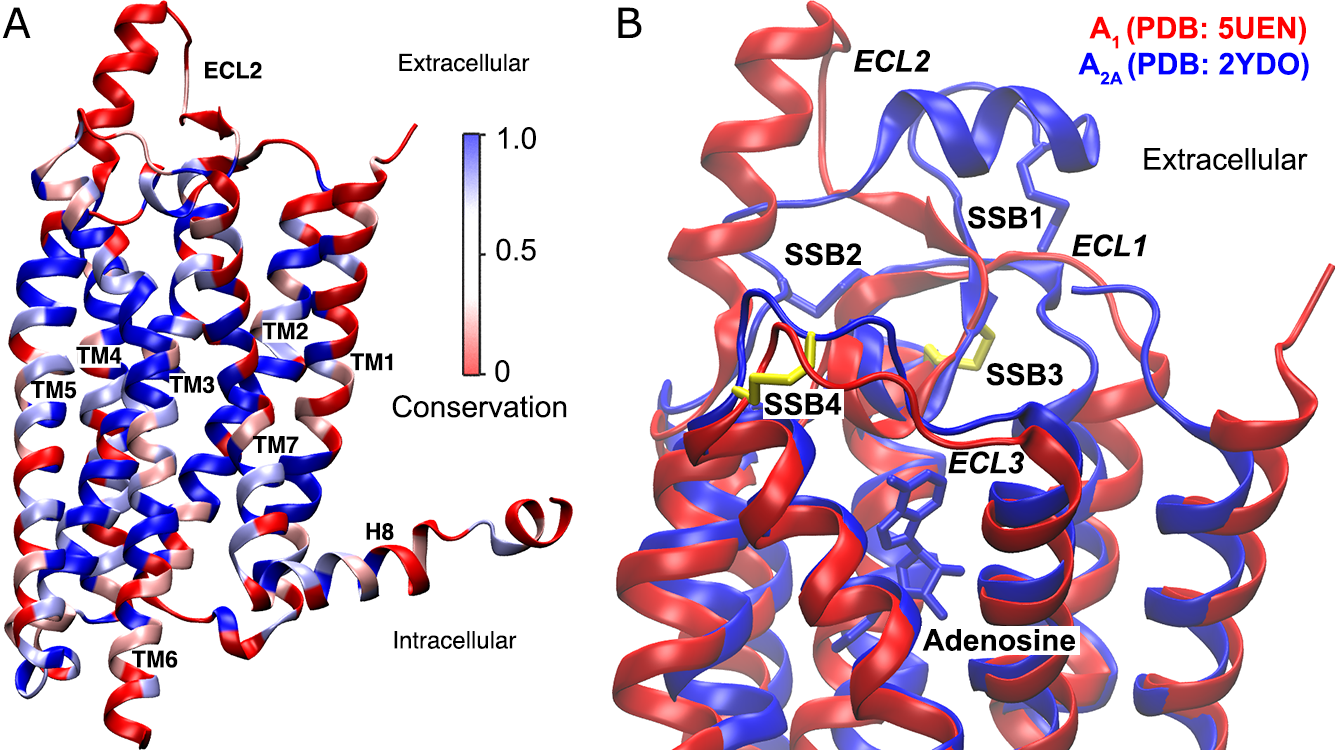


**Fig. S8** (A) Schematic representation of the A_1_AR colored by sequence conservation across four subtypes of ARs. A RWB color scale is used with 0 for low (red) and 1 for high (blue) conservation. (B) Comparison of X-ray structures of the A_1_AR (PDB: 5UEN, red) with the adenosine-bound A_2A_AR (PDB: 2YDO, blue). Two disulfide bonds, SSB1 and SSB2 that anchor ECL2 to ECL1 and Cys^3.22^ (blue sticks), are unique to A_2A_AR, although SSB3 and SSB4 that connect ECL2 to Cys^3.25^ and ECL3 to Cys^6.61^ (yellow sticks) are conserved.
